# Assessing methods for dealing with treatment switching in randomised controlled trials: a simulation study

**DOI:** 10.1186/1471-2288-11-4

**Published:** 2011-01-11

**Authors:** James P Morden, Paul C Lambert, Nicholas Latimer, Keith R Abrams, Allan J Wailoo

**Affiliations:** 1 ICR-CTSU, The Institute of Cancer Research, Sir Richard Doll Building, Cotswold Road, Sutton, Surrey, UK, SM2 5NG; 2 Centre for Biostatistics and Genetic Epidemiology, Department of Health Sciences University of Leicester, 2nd Floor Adrian Building, University Road, Leicester, UK, LE1 7RH; 3 Health Economics and Decision Science, ScHARR, University of Sheffield, Regent Court, 30 Regent Street, Sheffield, UK, S1 4DA

## Abstract

**Background:**

We investigate methods used to analyse the results of clinical trials with survival outcomes in which some patients switch from their allocated treatment to another trial treatment. These included simple methods which are commonly used in medical literature and may be subject to selection bias if patients switching are not typical of the population as a whole. Methods which attempt to adjust the estimated treatment effect, either through adjustment to the hazard ratio or via accelerated failure time models, were also considered. A simulation study was conducted to assess the performance of each method in a number of different scenarios.

**Results:**

16 different scenarios were identified which differed by the proportion of patients switching, underlying prognosis of switchers and the size of true treatment effect. 1000 datasets were simulated for each of these and all methods applied. Selection bias was observed in simple methods when the difference in survival between switchers and non-switchers were large. A number of methods, particularly the AFT method of Branson and Whitehead were found to give less biased estimates of the true treatment effect in these situations.

**Conclusions:**

Simple methods are often not appropriate to deal with treatment switching. Alternative approaches such as the Branson & Whitehead method to adjust for switching should be considered.

## Background

Randomized controlled trials (RCTs) are widely used to assess the effects of a new treatment or procedure compared to a control treatment. Survival outcomes are commonly used, particularly in the cancer setting, with the time to an event such as death or disease progression analysed. In advanced disease trials are often designed with progression free survival as the primary endpoint, and overall survival as a secondary endpoint.

It is common for patients to switch from the treatment to which they are randomised, either to the other trial treatment, a non-trial treatment or to stop receiving treatment altogether. Trial protocols often attempt to control these switches while maintaining a degree of flexibility over the treatment a patient can receive, although this varies greatly between trials, and switching remains common. Switches may occur for a number of reasons, many of which are related to an individual's prognosis. Most commonly patients will switch from the control arm to the intervention arm. A clinician may decide that a patient is responding poorly to their allocated treatment and it is therefore unethical to let them continue on this regime. Alternatively, a patient may switch because of treatment related side effects. Most often patients are allowed to switch to the new therapy at the point of disease progression. The estimate of the treatment effect on progression-free survival is unbiased if a patient's sole reason for switching treatments is their progression. However, the estimate of the treatment effect on overall survival is biased due to switching from control to the new therapy at progression. The question of interest is what would have been the overall survival treatment effect had no patients in the control arm switched?

Policy decisions (such as whether a health-care provider will fund a new treatment) increasingly rely on judgments of both clinical and cost effectiveness, both of which are heavily determined by the estimated survival gains of a technology. If there is crossover then individuals may receive treatments that are not consistent with the policy being evaluated. For example, if individuals switch from a standard treatment to a new treatment then they are not adhering to the policy of retaining standard care. Thus, failure to appropriately account for switching in deriving an estimate of treatment effect may lead to incorrect policy decisions and reduced efficiency of the health-care system as a whole.

An intention-to-treat (ITT) approach is often used where patients are analysed dependent on the treatment they are randomised to, regardless of whether they actually went on to receive this treatment for the entire follow-up period. This pragmatic approach is said to reflect the overall effectiveness of a treatment policy if it were introduced on a wider scale [1]. However, this is only the case where switching treatments is a feasible option. If the treatment is not currently available then treatment switching may not be an option in practice. It is often of interest to estimate the effectiveness of the experimental treatment alone, in the absence of switching. This appropriate policy effectiveness is especially important when assessing the cost-effectiveness of a treatment, something which is increasingly used as an input to drug reimbursement decisions [2].

Appropriate policy effectiveness is often quantified using a per-protocol (PP) approach which measures how well a patient fares dependent on the treatment they actually receive, regardless of which treatment arm they were randomised to. Patients who switch from their randomised treatment are therefore excluded from the analysis or censored at the time of their switch. This approach can lead to severe selection bias if those excluded differ in prognosis from those retained in the analysis, which is likely in this setting as patients often switch treatments because their condition has deteriorated [3].

The National Institute for Health and Clinical Excellence (NICE) has considered several drugs where crossover has been a feature of the key clinical trials. In the appraisal of trastuzumab for the treatment of metastatic breast cancer [4], 75% of patients randomised to control treatment in the key trial eventually switched to the experimental arm. These patients were excluded completely from the analysis and a median survival gain of 17.9 months was found. However, if all control patients had been included, this median survival gain was greatly reduced to just 7 months. The true median survival gain from the treatment is likely to be somewhere between these two values.

Crossover was also a feature of trials used in the recent appraisal of renal cell carcinoma therapies where the impact of alternative approaches on estimates of cost-effectiveness was highlighted [5]. For sunitinib, an analysis of overall survival which excluded all patients who received any subsequent therapy led to an Incremental Cost-Effectiveness Cost Ratio (ICER) of £59, 819 compared to standard care (interferon), based on a hazard ratio of 0.65. However if these patients were not excluded from the analysis, the overall hazard ratio is increased to 0.82, increasing the ICER to £118,005. In reality, the ICER is likely to lie somewhere in between these two estimates.

Various methods have been proposed to evaluate the appropriate policy effectiveness of a treatment taking into account deviations from the randomised treatment group. These range from relatively simple methods, such as per protocol analysis, to methods that account for switching using either a proportional hazards or accelerated failure time model. As it unclear how the various methods perform in different situations we evaluate them using a simulation study.

## Methods

The different approaches considered in this investigation can be grouped into simple methods (those which are currently widely used), and more sophisticated methods which make adjustments to the hazard ratio or use accelerated failure time models.

### Simple methods

Various methods have been used in existing literature in situations where patients depart from their randomised treatment. We refer to them here as simple methods which tend to involve only small adjustments to standard survival techniques. This section will focus on four of these, intention-to-treat, excluding or censoring patients if they switch treatments and modelling treatment as a time-varying covariate.

#### Intention-to-treat

Many authors take the pragmatic approach and use an intention-to-treat (ITT) analysis. Patients are analysed depending on which treatment arm they were randomised to. An important feature of an ITT analysis is that data from all randomised patients is utilised, with censoring used in time-to-event analysis for patients who are lost to follow-up to ensure this.

The results from an ITT analysis should always be given regardless of whether the effectiveness of the treatment is of interest as it reflects the design and conduct of the study. While analysis of this type is perfectly valid, it may underestimate the appropriate policy effectiveness of a treatment [6]. For example, if the experimental treatment truly is superior to the control treatment, and some patients have switched from control to experimental, and are therefore receiving the benefits of this, using an ITT analysis will make the treatments appear more similar than they really are. The benefit of this type of analysis is that randomisation balance between groups is maintained, reducing the possibility of bias affecting the results [7-9].

#### Per-protocol (excluding switchers or censoring at switch)

A per-protocol (PP) or as-treated approach involves analysing patients according to the treatment they actually received rather than that to which they were randomised. This is commonly used to supplement an intention-to-treat analysis [6].

Here we define a per-protocol approach as an attempt to estimate the policy effectiveness by censoring patients at the point at which they switch, or completely excluding any switching patients from the analysis. Therefore unlike the ITT analysis described previously, not all available patient data is utilised. Whereas ITT uses randomisation to ensure treatment arms are balanced in all aspects other than that of treatment, PP analysis may be subject to selection bias as groups may no longer be balanced after a patient is censored or excluded [7]. This type of bias is particularly likely if a patient's probability of switching treatments is strongly related to their underlying prognosis [10].

#### Treatment as a time-varying covariate

An extension of the Cox proportional hazards model is to include treatment as a time-varying covariate to assess the effect of treatment actually received by a patient. The model takes the form:

(1) $$\lambda_{i}\left(t\right)=\lambda_{0}\left(t\right)\exp\left(\beta X_{i}\left(t\right)\right)$$

where *λ*_0_(*t*) is the baseline hazard function and *X_i_*(*t*) takes a value of zero while a patient is receiving the control and 1 while they are receiving the experimental treatment. However, like the PP methods presented above, this method can break the randomisation balance and is therefore subject to selection bias if switching is related to prognosis [11].

### Adjusted hazard ratio methods

The two methods described here make adjustments to the hazard ratio in order to take into account patients switching from one treatment arm to the other.

#### Adjusted Cox model (Law and Kaldor, 1996)

Law and Kaldor [12] propose a method of adjusting the standard Cox model to take into account patients who depart from their randomised treatment. The method can be used in situations where patients switch both from control to experimental treatment and/or in the opposite direction.

The method works on the principle that patients can be divided into four groups depending on their switching pattern. So given an RCT comparing two treatments (*A *and *B*), patients are classified as being in Group *AA *or *BB *if they were allocated to *A *or *B *and did not switch treatments, or to Group *AB *or *BA *if they switched from their allocated treatment to the other treatment. The hazard rates in each group are assumed proportional. A Cox model is then fitted with a time-varying covariate for switching time. Full details of the method can be found in the paper itself [12].

The method makes a number of assumptions which may not be appropriate in all situations. The assumption that the underlying hazard rates of switchers and non-switchers allocated to each treatment can be expressed as multiplicative factors may not be appropriate and is not testable. Also, it is assumed that switching onto a new treatment will cause an instantaneous improvement, which may be important, but would be difficult to test in reality. The method also makes the assumption that the treatment effect for patients switching on to a treatment will be the same as for those initially allocated to receive it. This assumption is unlikely to be true in a real trial setting for a number of reasons, the most important of which may be that patients switching onto a treatment will typically be at a more advanced stage of their disease than those in the treatment arm were at the start of the trial. This assumption could be tested for a real dataset by comparing the survival times of patients from their switch with the survival times of treatment arm patients, although analysis of this type would itself be subject to bias.

Problems with this method have been raised by White [13]. Patients are grouped as described above according to future events, i.e. a treatment switch which has not yet occurred. So for example, subjects in group *AB *are said to have a certain hazard function before they switch. However, in reality they have a hazard of zero up to the point at which they switch treatment, as they cannot die before this point or they would be in group *AA*. White states that this is likely to bias the estimated hazard ratio towards the null.

#### Causal proportional hazards estimator (Loeys and Goetghebeur, 2003)

Loeys and Goethebeur [14] present a method for calculating the true treatment efficacy in situations where all patients take their allocated treatment in one arm and compliance is "all-or-nothing" in the other arm. This means that if a patient in this arm switches, the switch is assumed to have happened at time zero, and the patient is assumed to have only received the treatment they switched onto and none of their allocated treatment. The method and its implementation in the Stata package are described further by Kim and White [15].

The authors consider a clinical trial in which patients are randomised to receive either a control treatment or an experimental treatment. The method works on the assumption that all patients in the control arm comply fully, and patients in the experimental arm may either comply fully (complier) or not at all (non-complier). Patients in the control arm are also classed as either being a complier or non-complier depending on how they would have behaved *if *they had been randomised to the experimental arm. The proportion of non-compliers is assumed to be the same in both arms due to randomisation (often referred to as the exclusion restriction [16]).

The method then makes use of Kaplan-Meier survival estimates and the assumed relationship between control and experimental compliers to find an estimate of the hazard ratio. Loeys and Goetghebeur [14] give full details of the methodology used. The method was applied in this investigation using the Stata program *stcomply *as described by Kim and White [15,17].

The all-or nothing compliance assumption is a very important limitation of this method as this type of compliance is only likely to occur in very specific scenarios, such as a trial to investigate a new screening program where patients may be allocated to attend screening but may not attend. As mentioned previously the method also makes the important exclusion restriction assumption, which although untestable, we feel is likely to hold in most settings. The implications of a violation of this assumption has been discussed elsewhere [16,18], and may be reduced by incorporating covariates that predict compliance.

### Accelerated failure time model methods

The methods in this section make use of accelerated failure time (AFT) models, an alternative form of survival model to the commonly used proportional hazards model. A proportional hazards model assumes that covariates multiply the hazard by a constant, whereas an AFT model assumes that a covariate multiplies the predicted event time by a constant [19].

These methods have been referred to as randomisation-based efficacy estimators (RBEEs) [6], as they compare groups as randomised and therefore are intended to reduce biases which may be introduced by comparing groups as-treated. The method of Loeys & Goetghebeur [14] described previously is also an RBEE as it preserves the randomisation balance and the significance level from an ITT analysis.

#### Rank preserving structural failure time models (Robins and Tsiatis, 1991)

Robins and Tsiatis [20] describe the use of AFT models to estimate the true efficacy of a treatment. A patient's observed event time is related to their *counterfactual *event time, that which would have been observed for a particular patient if they had not received any treatment. These models are referred to as rank preserving as they make the assumption that given two patients *i *and *j*, if *i *failed before *j *when both were on one treatment, then *i *would also fail before *j *if both patients took the same alternative treatment. Consider a randomised trial with two arms, a control arm (*A*) receiving no treatment, and an experimental arm (*B*). Each patient *i *has an observed time to event or censoring *T_i_*. *R_i _*= *A *or *B *is the patient's randomised treatment arm.

Each patient also has a counterfactual event time *U_i _*which is the event time which would have been observed if no treatment had been received. Patients in the control arm who do not switch treatment will have *T_i _*= *U_i_*, so their counterfactual event time will be observed. *U_i _*is unobserved for all other patients. The assumption is made that *U_i _*is independent of *R_i _*due to randomisation balance.

Consider the observed event time *T_i _*as being made up of a patient's time on the control treatment *T_Ai _*and their time on the experimental treatment *T_Bi_*, so *T_i _*= *T_Ai _*+ *T_Bi_*. For patients who did not switch treatments, either *T_Ai _*or *T_Bi _*will be equal to zero. *T_i _*is related to the counterfactual event time *U_i _*by the following causal model:

(2) $$U_{i}=T_{Ai}+e^{-\psi_{0}}T_{Bi}$$

$e^{\psi_{0}}$ is often called the *acceleration factor*, the amount by which a patient's expected time to event is increased by treatment. A value of $e^{\psi_{0}}>1$ indicates a beneficial treatment effect whereas $e^{\psi_{0}}<1$ suggests treatment has a detrimental effect, increasing the speed at which a patient moves towards their event. $e^{\psi_{0}}$ is perhaps easier to interpret than $e^{-\psi_{0}}$ so results will be presented in this form.

By defining a binary process *X_i_*(*t*) which equals 1 when a patient is on experimental treatment and 0 otherwise, equation (2) can be rewritten as:

(3) $$U_{i}=\int_{0}^{T_{i}}\exp\left[\psi X_{i}\left(t\right)\right]\;dt$$

For a given value of *ψ*, the hypothesis *ψ_0 _= ψ *can be tested by first calculating *U_i_*(*ψ*) using equation (2). *Z*(*ψ*) is then calculated as the test statistic for the hypothesis *U*(*ψ*)╨*R*, i.e. a patient's counterfactual event time is independent of the treatment arm to which they were randomised.

A number of different tests could be used to calculate *Z*(*ψ*). We considered four different statistical tests in this investigation: the logrank, Cox, exponential and Weibull tests. The value of *ψ *for which *Z*(*ψ*) = 0 is taken as the point estimate. This is the value for which *U *is balanced between treatment arms.

The method has been extended and implemented in Stata (through the *strbee *program) by White et al [21,22] as follows. Define *C_i _*as the administrative censoring time which corresponds to the end of follow-up. Using equation (3), the censoring time for *U_i_*(*ψ*) is given by:

(4) $$D_{i}\left(\psi \right)=\int_{0}^{C_{i}}\exp\left[\psi X_{i}\left(t\right)\right]\;dt$$

However, because switching may be related to prognosis, both *X_i _*and *D_i _*may also depend on prognosis and therefore the censoring of *U_i_*(*ψ*) may be informative, and thus including censoring in this way can result in biased estimates as shown by White et al [22]. They suggest this bias could be avoided by recensoring counterfactual survival times so that the censoring time equals the minimum of the administrative censoring time (*C_i_*) and *C_i _*exp *ψ*. Then the counterfacutal survival time *U_i_*(*ψ*) is replaced by the censoring time of the counterfactual event times $D_{i}^{*}\left(\psi \right)$ if $D_{i}^{*}\left(\psi \right)<U_{i}\left(\psi \right)$.

An interval bisection process can be used to find the point estimate and confidence interval for *ψ*. Further details of this can be found in the discussion of the *strbee *program [21].

The Robins & Tsiatis method makes a number of assumptions. As mentioned previously the models are rank-preserving, which may not be plausible with certain patients likely to see more or less benefit than others on different types of treatments due to biological factors. However testing for any violations of this assumption in real data may not be possible. The method also assumes an equal treatment effect for patients switching to a treatment as for those initially allocated to receive it as discussed previously for the Law & Kaldor method

#### Iterative parameter estimation algorithm (Branson and Whitehead, 2002)

Branson and Whitehead [23] build on the method developed by Robins and Tsiatis [20] by replacing the test-based estimation of *ψ *with a likelihood-based analysis.

An iterative parameter estimation (IPE) algorithm is used. This retains all patients to the treatment group to which they were initially randomised. Using the same notation as used in the previous section, consider the model relating counterfactual and observed event times seen previously.

An initial estimate for *e^ψ ^*is obtained by comparing the treatment arms as randomised using an parametric failure time model (equivalent to an intention-to-treat approach). A number of parametric distributions could be chosen for this such as log-logistic, log-normal or gamma. We use a Weibull distribution as it has the advantage of having both AFT model and proportional hazards model parameterisations [19].

Given this initial estimate, the observed survival times of patients who switched from control to experimental treatment are transformed using the current estimate for *e^ψ ^*and equation (2). Groups are compared again, giving an updated estimate for *e^ψ^*. The process is then repeated until the latest value of *e^ψ ^*becomes sufficiently close to its value from the previous iteration, at which point the process is said to have converged. Further explanation of the algorithm can be found in the original paper [23].

If the algorithm projects a patient's survival time beyond the administrative censoring time *C_i_*, the patient is considered censored and their projected survival time is replaced by *C_i_*. This recensoring is restricted only to patients in the control arm who switch treatments, unlike the recensoring implemented to the Robins and Tsiatis method by White et al [22].

Standard errors can be calculated by either taking the standard error from the final regression in the algorithm or by using bootstrapping [24]. The authors discuss how the standard error from the final regression may be too small meaning bootstrapping may be preferable. This is because the covariance matrix from the final iteration of the IPE does not does not take into account the fact that control arm patients have had their survival time adjusted by the algorithm.

This method makes all the assumptions of the Robins and Tsiatis method, and in addition assumes that survival times take a certain parametric form (although the relationship between a patient's prognosis and their switching pattern is still not modelled). This is an important additional assumption, with a violation having a potential impact on the estimation of an adjusted treatment effect. The authors suggest that given a real dataset, a parametric form is chosen which fits the observed data most closely.

#### Parametric randomisation-based methods (Walker et al, 2004)

In the previous two methods *ψ *is chosen to balance the counterfactual event time, *U*, between treatment arms. However as discussed previously, and by Robins and Tsiatis [20], these methods can be associated with a loss of information through recensoring and arbitrary differences from the results of ITT analysis. Walker et al [25] present an extension to these semi-parametric methods which involve full parametric modelling of the relationship between *U *and the treatment a patient actually receives *Z*. Again we consider a trial with control (*A*) and experimental (*B*) arms where some patients who are randomised to control actually switch to receive the experimental treatment at some point during follow-up. Consider *U_i _*as a patient's counterfactual event time and *Z_i _*as the time at which they start receiving experimental treatment. The authors propose specifying a joint parametric model for *U_i _*and *Z_i _*which is made up of three parts:

1. **A causal model relating ***U_i _***to a patient's observed failure time ***T_i_*. This is the AFT model seen in previous sections.

2. **A model for the association between ***U ***and ***Z*. This is a bivariate frailty model. Either a positive stable [26] or gamma [27] frailty are suggested. These models include a parameter *ϕ *which describes the level of association between *U *and *Z*.

3. **Models for the marginal cumulative hazards**. *H_u_*(*u*) and *H_z_*(*z*) are the marginal cumulative hazards of *U *and *Z *respectively.

Fitting this model using maximum-likelihood techniques would only ensure the original randomisation balance is preserved if all models are correctly specified. Parameter estimates will therefore be very sensitive to inaccuracies in the model specification. To deal with this, the authors suggest an alternative approach to maximum likelihood to estimate parameters. They use an augmented model to maintain the randomisation balance between groups which corresponds to the Cox model based test statistic in the semi-parametric approach of Robins & Tsiatis. The model has the form:

(5) $$H_{u}^{*}\left(u\right)=e^{\rho R}H_{u}\left(u\right)$$

An estimate of *ψ *can be found so that an estimate of *ρ *would be equal to zero, indicating there is no relationship between a patient's underlying survival time and the treatment arm they are randomised to so randomisation balance is maintained. Full details of the estimation process are described by Walker et al [25]. The method is implemented here through the *gparmee *program in Stata [17].

This method makes the same assumptions as the Robins & Tsiatis method, and in addition makes assumptions about the parametric form of the data. The authors suggest distributions chosen could be based on the observed data, although choosing an appropriate frailty distribution could be difficult. However the authors suggest that the method is robust to model misspecifications when the estimating equations approach is used.

### Simulation study design

To formally assess the various methods, a simulation study was conducted. Independent datasets were simulated with the true difference between each treatment's effect on survival known and each method applied to the data to see how well they performed in terms of bias, variability and coverage. The simulated data was designed to reflect data which is obtained from real clinical trials based on a review of recent submissions to NICE. This section contains details of the design of the simulation study.

#### Underlying survival times

The starting point for simulating data was to generate a number of patients with an underlying survival time. A sample size of 500 was chosen, with 250 patients allocated each to receive control or experimental treatment. This sample size reflects what is often seen in large cancer trials [4,28,29]. Survival times for these patients were then generated from a Weibull distribution as described by Bender et al [30]. The shape parameter *γ *was set at 0.5 which assumes mortality rate is decreasing over time, a situation often observed in cancer data [31,32]. The scale parameter *λ *was chosen so that approximately 90% of patients who receive no treatment had died after three years of follow-up.

#### Entry and exit times

Patients were assumed to have entered the study at some point during a one-year period, with their entry time generated from a uniform distribution between time zero and 1 year. Patients were then censored at 3 years to represent the end of the follow-up period. Therefore all patients were followed up for between 2 and 3 years, dependent on their entry time, representing what is often seen in a real trial setting.

#### Patient prognosis

As described previously, bias can often occur when patients with different underlying prognoses have different probabilities of switching between treatment arms. To investigate this, patients were split into two groups, those with a 'good' prognosis and those with a 'poor' prognosis. The probability of a patient being in the 'good' prognosis group was set at either 30% or 75%. Patients allocated to the 'good' prognosis group were assumed to have their previously generated underlying survival time multiplied by an inflation factor. Values of 1.2 and 3 were chosen to represent relatively small and large differences between the prognostic groups. Randomisation should ensure the proportion with 'good' and 'bad' prognosis was balanced between treatment arms.

#### Switching probability

The probability of a patient switching was then set, dependent on their prognosis group. Only switching from the control to the experimental treatment was considered. The assumption was made that patients in the 'poor' prognosis group were more likely to crossover, as is often the case with the experimental treatment considered as a "rescue" measure. Two sets of probabilities were considered; probabilities of switching 10% and 25% for 'good' and 'poor' prognosis groups respectively to represent a relatively small proportion of patients switching treatments or 50% and 75% for 'good' and 'poor' groups respectively to represent a trial with a large proportion of control patients switching. These probabilities were then used to generate a binary variable indicating whether or not a patient switches treatments.

#### Switching time

For patients who switched treatments, a switching time was generated which occurred between their entry into the study and their exit (through either death or censoring). Switching times were generated using a uniform distribution. This assumes that a patient is equally likely to switch at any point between their entry into the study and death or censoring.

#### Adjusting survival times for treatment received

The next step is to adjust survival times based on the amount of treatment a patient actually receives. For each patient, survival time is made up of time on control *T_Ai _*and time on experimental treatment *T_Bi_*. Patients randomised to control who do not switch treatments will have *T_Bi _*= 0. All patients randomised to experimental treatment will have *T_Ai _*= 0 as no patients from this arm are allowed to switch treatments. Adjusted patient survival time $T_{i}^{*}$ is then calculated using the formula for the causal accelerated failure time model as described by Walker et al [25]:

(6) $$T_{i}^{*}=T_{Ai}+e^{\psi }T_{Bi}$$

where *e^ψ ^*is the true effect of treatment. Patient times are therefore extended beyond the time that they spend on control. If a patient's survival time is extended beyond three years they are censored at three years.

#### Treatment effect

Initial treatment effect hazard ratios of 0.9 and 0.7 were chosen to represent situations with a smaller and larger true difference between treatments, with the experiment treatment considered beneficial.

As the values *λ *of and *γ *used to simulate the underlying survival times are known, the hazard ratios *β *described above can be converted into *e^ψ ^*form required by equation (6) by using the formula described by Collett [19]:

(7) $$\psi =\frac{-\ln\beta }{\gamma }$$

For example, a hazard ratio of 0.7, with *γ *= 0.5 equates to *ψ *= 0.7133 and therefore *e^ψ ^*= 2.04.

Table 1 gives a summary of all variables considered when simulating patient data and the values chosen for these.

**Table 1:** Summary of simulation variables

Variable	Scenarios	Details
Sample size	1	500 patients, 250 in each treatment arm

Weibull shape parameter *γ*	1	0.5, to represent mortality rate decreasing over time

Weibull scale parameter *λ*	1	1.33, chosen such that 90% of patients have died after 3 years of follow-up

Probability of patient having 'good' prognosis	2	30% or 75%

Difference in survival between 'good' and 'poor' Prognosis groups	2	Survival times of 'good' prognosis group multiplied by a factor of either 1.2 or 3

Probability of switching treatment dependent on prognosis group	2	10% ('good' prognosis) and 25% ('poor' prognosis) or 50% ('good' prognosis) and 75% ('poor' prognosis)

Switching time	1	Generated from a Uniform distribution

Initial treatment effect	2	Hazard ratio of 0.9 or 0.7

#### Applying the methods

By considering all possible combinations of the variables described in Table 1, 16 scenarios were identified. For each of these, data was generated as described above, and the various methods applied to this dataset. This process was repeated 1000 times for each scenario. For each method the mean treatment effect $\bar{\hat{\beta }}$ and its standard error *SE*($\hat{\beta }$) over the 1000 simulations were calculated. The means of the standard error and 95% confidence limits from each method were also calculated. No standard errors are given by the Loeys & Goetghebeur or Robins & Tsiatis methods. For the Branson & Whitehead method, standard errors were taken from the final regression of the algorithm rather than bootstrapping due to the large computing time bootstrapping for each simulated dataset would require.

#### Performance measures

Measures which can be used to assess the methods presented were calculated as described by Burton et al [33]. The bias of each method *δ *was calculated as:

(8) $$\delta =\bar{\hat{\beta }}-\beta$$

where *β *is the true initial treatment effect for that particular scenario.

The mean square error (MSE) is a useful measure of the overall accuracy of a method as it includes both measures of bias and of the variability of estimates given by a method [33]. The MSE is calculated as:

(9) $$MSE={\left(\bar{\hat{\beta }}-\beta \right)}^{2}+{\left(SE\left(\hat{\beta }\right)\right)}^{2}$$

Also calculated was the coverage of each method. This is the defined as the proportion of times the 95% confidence interval for a particular method contains the true treatment effect, *β*. Coverage should be approximately equal to 95%, indicating that around 95% of the confidence intervals include the true value. As some methods may not successfully converge in certain situations, the proportion of times each method successfully gave a parameter estimate was also calculated. Methods which are unsuccessful for a large number of simulated datasets may be of little practical use.

## Results

Table 2 shows details of the parameter values used in each of the 16 scenarios and the table in which the results for this scenario can be found. Results from scenarios 3, 4, 7, 8, 11, 12, 15 and 16 can be found in [Additional files 1, 2, 3 and 4]. A selection of results are presented in this section.

**Table 2:** List of scenarios

Scenario Number	Treatment effect (HR)		% with 'good' Prognosis		'Good' prognosis Survival		Crossover probabilities ('good' and 'poor' prognosis)		Table Number
	0.9	0.7	30%	75%	×1.2	×3	10% and 25%	50% and 75%	
1	√		√		√		√		3
2		√	√		√		√		3
3	√			√	√		√		Additional file 1
4		√		√	√		√		Additional file 1
5	√		√		√			√	4
6		√	√		√			√	4
7	√			√	√			√	Additional file 2
8		√		√	√			√	Additional file 2
9	√		√			√	√		5
10		√	√			√	√		5
11	√			√		√	√		Additional file 3
12		√		√		√	√		Additional file 3
13	√		√			√		√	6
14		√	√			√		√	6
15	√			√		√		√	Additional file 4
16		√		√		√		√	Additional file 4

For figures in this section, method names were abbreviated as follows: Intention-to-Treat (ITT), Exclude switchers (PP-EXC), Censor at switch (PP-CENS), Treatment as time-varying covariate (TVC), Law & Kaldor (LK), Loeys & Goetghebeur (LG), Robins & Tsiatis with logrank test (RT-LR), with Cox test (RT-COX), with exponential test (RT-EXP), with Weibull test (RT-WB), Branson & Whitehead (BW) and Walker et al parametric method (WALK).

### Prognosis and bias

We will first focus on four particular scenarios, 2, 6, 10 and 14. Each of these has 30% of patients with 'good' prognosis, a true treatment difference of *β *= 0.7 on the hazard ratio scale or *e^ψ ^*= 2.04 on the AFT scale.

The scenarios vary in the difference in survival between 'good' and 'poor' prognosis groups, with 'good' prognosis patient's survival multiplied by 1.2 in scenarios 2 and 6 and by 3 in scenarios 10 and 14. The scenarios also differ in the probabilities of switching in 'good' and 'poor' prognosis groups, with probabilities of 10% and 25% respectively in scenarios 2 and 10 and of 50% and 75% respectively in scenarios 6 and 14. Full results from these scenarios can be found in Tables 3, 4, 5 and 6.

**Table 3:** Scenarios 1 & 2: 30% 'good' prognosis, ×1.2 'good' prognosis survival, 10% and 25% switching probabilities, 100% treatment effect for switchers, uniform distribution for switching times

True HR and *e^ψ^*	Method	Mean estimate	Mean SE	SE of mean	95% Confidence interval		Bias	MSE	Coverage (%)	Successful estimation (%)
					Lower	Upper				
	**Hazard ratio methods**									
	ITT	0.9120	0.0884	0.0882	0.7542	1.1027	0.0120	0.0079	94.6	100.0
	PP - Exclude switchers	0.9053	0.0932	0.0929	0.7399	1.1076	0.0053	0.0087	94.3	100.0
	PP - Censor at switch	1.0589	0.1091	0.1086	0.8653	1.2957	0.1589	0.0370	67.5	100.0
	Time-dependent covariate	1.1998	0.1189	0.1211	0.9880	1.4570	0.2998	0.1046	19.1	100.0
	Law and Kaldor	0.9168	0.1136	0.1132	0.7192	1.1688	0.0168	0.0131	94.7	100.0
	Loeys and Goethebeur	0.8900	-	0.1086	0.6999	1.1354	-0.0100	0.0119	94.8	100.0
0.9 & 1.23	**AFT methods**									
	ITT	1.2357	0.2392	0.2423	0.8457	1.8060	0.0011	0.0587	94.6	100.0
	PP - Exclude switchers	1.2586	0.0932	0.2616	0.8415	1.8830	0.0240	0.0690	94.3	100.0
	PP - Censor at switch	0.9214	0.1864	0.1897	0.6199	1.3700	-0.3132	0.1341	66.3	100.0
	Robins and Tsiatis - Logrank	1.2715	-	0.2852	0.8244	1.9604	0.0370	0.0827	94.7	100.0
	Robins and Tsiatis - Cox	1.2703	-	0.2806	0.8199	1.9888	0.0357	0.0800	95.2	97.2
	Robins and Tsiatis - Exponential	1.2781	-	0.2820	0.9712	1.7776	0.0436	0.0814	83.0	99.7
	Robins and Tsiatis - Weibull	1.2714	-	0.2845	0.8278	1.9933	0.0369	0.0823	95.0	99.7
	Branson and Whitehead	1.2681	0.2455	0.2745	0.8678	1.8536	0.0335	0.0765	92.7	100.0
	Walker et al	2.2108	1.1659	1.1869	0.8323	1218.7190	0.9763	2.3617	76.6	99.4

	**Hazard ratio methods**									
	ITT	0.7315	0.0731	0.0743	0.6014	0.8897	0.0315	0.0065	93.6	100.0
	PP - Exclude switchers	0.7050	0.0744	0.0776	0.5733	0.8669	0.0050	0.0060	94.3	100.0
	PP - Censor at switch	0.8215	0.0868	0.0886	0.6678	1.0106	0.1215	0.0226	69.2	100.0
	Time-dependent covariate	0.9364	0.0950	0.0982	0.7675	1.1424	0.2364	0.0655	18.8	100.0
	Law and Kaldor	0.7376	0.0957	0.0968	0.5720	0.9511	0.0376	0.0108	93.6	100.0
	Loeys and Goethebeur	0.6733	-	0.0876	0.5220	0.8672	-0.0267	0.0084	93.2	100.0
0.7 & 2.04	**AFT methods**									
	ITT	1.9259	0.3858	0.3992	1.3007	2.8524	-0.1150	0.1726	93.5	100.0
	PP - Exclude switchers	2.0825	0.0744	0.4676	1.3766	3.1516	0.0417	0.2204	94.3	100.0
	PP - Censor at switch	1.5194	0.3140	0.3241	1.0136	2.2786	-0.5214	0.3769	66.5	100.0
	Robins and Tsiatis - Logrank	2.1014	-	0.5024	1.3483	3.3204	0.0606	0.2561	94.5	100.0
	Robins and Tsiatis - Cox	2.0969	-	0.4977	1.3303	73.7821	0.0561	0.2508	94.9	93.5
	Robins and Tsiatis - Exponential	2.1041	-	0.5090	1.4957	3.1128	0.0633	0.2631	87.1	100.0
	Robins and Tsiatis - Weibull	2.1017	-	0.5024	1.3497	3.4566	0.0609	0.2561	94.9	100.0
	Branson and Whitehead	2.0889	0.4188	0.4770	1.4104	3.0949	0.0481	0.2299	92.2	100.0
	Walker et al	3.6507	1.8935	1.5878	1.3852	92.4403	1.6099	5.1127	79.5	93.1

**Table 4:** Scenarios 5 & 6: 30% 'good' prognosis, ×1.2 'good' prognosis survival, 50% and 75% switching probabilities, 100% treatment effect for switchers, uniform distribution for switching times

True HR and *e^ψ^*	Method	Mean estimate	Mean SE	SE of mean	95% Confidence interval		Bias	MSE	Coverage (%)	Successful estimation (%)
					Lower	Upper				
	**Hazard ratio methods**									
	ITT	0.9364	0.0909	0.0909	0.7741	1.1328	0.0364	0.0096	93.6	100.0
	PP - Exclude switchers	0.9204	0.1275	0.1278	0.7016	1.2074	0.0204	0.0167	96.0	100.0
	PP - Censor at switch	2.1663	0.3018	0.2975	1.6488	2.8466	1.2663	1.6921	0.0	100.0
	Time-dependent covariate	3.0639	0.4065	0.4054	2.3625	3.9740	2.1639	4.8466	0.0	100.0
	Law and Kaldor	0.9396	0.1168	0.1144	0.7364	1.1987	0.0396	0.0147	94.2	100.0
	Loeys and Goethebeur	0.8435	-	0.2725	0.4578	1.8486	-0.0565	0.0774	92.8	99.9
0.9 & 1.23	**AFT methods**									
	ITT	1.1732	0.2280	0.2311	0.8018	1.7173	-0.0613	0.0572	93.2	100.0
	PP - Exclude switchers	1.2492	0.1275	0.3479	0.7276	2.1475	0.0146	0.1212	95.9	100.0
	PP - Censor at switch	0.2373	0.0665	0.0663	0.1371	0.4115	-0.9973	0.9989	0.0	100.0
	Robins and Tsiatis - Logrank	1.2882	-	0.3862	0.6906	2.2552	0.0536	0.1521	93.8	100.0
	Robins and Tsiatis - Cox	1.2855	-	0.3874	0.6868	2.2531	0.0509	0.1527	93.9	96.3
	Robins and Tsiatis - Exponential	1.3036	-	0.3693	0.9172	1.9617	0.0691	0.1412	83.6	100.0
	Robins and Tsiatis - Weibull	1.2879	-	0.3857	0.6988	2.2550	0.0534	0.1516	94.5	100.0
	Branson and Whitehead	1.2841	0.2497	0.3619	0.8772	1.8802	0.0495	0.1334	83.7	100.0
	Walker et al	2.1037	0.7802	0.7791	1.0276	4.4146	0.8692	1.3624	70.0	99.8

	**Hazard ratio methods**									
	ITT	0.8073	0.0812	0.0814	0.6629	0.9833	0.1073	0.0181	71.5	100.0
	PP - Exclude switchers	0.7179	0.1009	0.1019	0.5451	0.9457	0.0179	0.0107	94.8	100.0
	PP - Censor at switch	1.6825	0.2388	0.2454	1.2740	2.2223	0.9825	1.0256	0.0	100.0
	Time-dependent covariate	2.4211	0.3257	0.3396	1.8602	3.1516	1.7211	3.0776	0.0	100.0
	Law and Kaldor	0.8110	0.1064	0.1046	0.6271	1.0488	0.1110	0.0233	81.2	100.0
	Loeys and Goethebeur	0.5248	-	0.1728	0.2544	1.0233	-0.1752	0.0606	83.8	99.0
0.7 & 2.04	**AFT methods**									
	ITT	1.5845	0.3208	0.3294	1.0657	2.3569	-0.4563	0.3167	72.2	100.0
	PP - Exclude switchers	2.0610	0.1009	0.6062	1.1886	3.5793	0.0202	0.3679	94.4	100.0
	PP - Censor at switch	0.3894	0.1088	0.1126	0.2254	0.6742	-1.6514	2.7398	0.0	100.0
	Robins and Tsiatis - Logrank	2.1120	-	0.6624	1.1204	3.8159	0.0712	0.4439	94.8	100.0
	Robins and Tsiatis - Cox	2.1062	-	0.6612	1.1095	3.8198	0.0654	0.4415	95.1	92.6
	Robins and Tsiatis - Exponential	2.1121	-	0.6636	1.3323	3.3923	0.0713	0.4455	87.4	100.0
	Robins and Tsiatis - Weibull	2.1128	-	0.6631	1.1249	3.8301	0.0719	0.4449	94.9	100.0
	Branson and Whitehead	2.0536	0.4177	0.6096	1.3787	3.0601	0.0127	0.3718	83.0	100.0
	Walker et al	3.6581	1.3293	1.2498	1.8098	7.5537	1.6172	4.1774	63.9	98.0

**Table 5:** Scenarios 9 & 10: 30% 'good' prognosis, ×3 'good' prognosis survival, 10% and 25% switching probabilities, 100% treatment effect for switchers, uniform distribution for switching times

True HR and *e^ψ^*	Method	Mean estimate	Mean SE	SE of mean	95% Confidence interval		Bias	MSE	Coverage (%)	Successful estimation (%)
					Lower	Upper				
	**Hazard ratio methods**									
	ITT	0.9136	0.0911	0.0926	0.7515	1.1107	0.0136	0.0088	94.8	100.0
	PP - Exclude switchers	0.9235	0.0979	0.0968	0.7504	1.1367	0.0235	0.0099	94.6	100.0
	PP - Censor at switch	1.0721	0.1137	0.1128	0.8709	1.3197	0.1721	0.0424	63.7	100.0
	Time-dependent covariate	1.2253	0.1249	0.1254	1.0035	1.4962	0.3253	0.1215	15.2	100.0
	Law and Kaldor	0.9121	0.1173	0.1223	0.7088	1.1737	0.0121	0.0151	94.1	100.0
	Loeys and Goethebeur	0.8933	-	0.1137	0.6975	1.1444	-0.0067	0.0130	95.3	100.0
0.9 & 1.23	**AFT methods**									
	ITT	1.2399	0.2523	0.2519	0.8323	1.8479	0.0054	0.0635	94.7	100.0
	PP - Exclude switchers	1.2159	0.0979	0.2580	0.7959	1.8583	-0.0187	0.0669	94.9	100.0
	PP - Censor at switch	0.8983	0.1911	0.1890	0.5922	1.3632	-0.3363	0.1488	64.4	100.0
	Robins and Tsiatis - Logrank	1.2838	-	0.3018	0.8085	2.0389	0.0492	0.0935	94.6	100.0
	Robins and Tsiatis - Cox	1.2854	-	0.3020	0.8073	2.0820	0.0508	0.0938	94.7	95.2
	Robins and Tsiatis - Exponential	1.2895	-	0.2934	0.9593	1.8561	0.0549	0.0891	84.8	99.8
	Robins and Tsiatis - Weibull	1.2836	-	0.3003	0.8153	2.0802	0.0490	0.0926	94.5	99.8
	Branson and Whitehead	1.2713	0.2589	0.2823	0.8530	1.8954	0.0368	0.0810	92.9	100.0
	Walker et al	2.9706	1.4458	1.4409	1.1904	20.7004	1.7360	5.0898	57.0	98.6

	**Hazard ratio methods**									
	ITT	0.7390	0.0760	0.0777	0.6041	0.9041	0.0390	0.0076	92.0	100.0
	PP - Exclude switchers	0.7267	0.0791	0.0800	0.5872	0.8995	0.0267	0.0071	93.8	100.0
	PP - Censor at switch	0.8418	0.0918	0.0938	0.6798	1.0422	0.1418	0.0289	60.1	100.0
	Time-dependent covariate	0.9698	0.1012	0.1054	0.7904	1.1899	0.2698	0.0839	14.8	100.0
	Law and Kaldor	0.7397	0.0998	0.1027	0.5679	0.9637	0.0397	0.0121	92.8	100.0
	Loeys and Goethebeur	0.6840	-	0.0908	0.5243	0.8886	-0.0160	0.0085	95.5	100.0
0.7 & 2.04	**AFT methods**									
	ITT	1.9110	0.4010	0.4163	1.2669	2.8838	-0.1298	0.1901	92.5	100.0
	PP - Exclude switchers	1.9836	0.0791	0.4501	1.2841	3.0655	-0.0573	0.2059	94.7	100.0
	PP - Censor at switch	1.4612	0.3165	0.3284	0.9560	2.2344	-0.5796	0.4438	59.4	100.0
	Robins and Tsiatis - Logrank	2.1150	-	0.5430	1.3172	3.4305	0.0742	0.3004	94.7	100.0
	Robins and Tsiatis - Cox	2.1140	-	0.5395	1.2935	27.2997	0.0732	0.2964	94.9	92.2
	Robins and Tsiatis - Exponential	2.1112	-	0.5439	1.4578	3.1966	0.0704	0.3008	87.4	100.0
	Robins and Tsiatis - Weibull	2.1150	-	0.5434	1.3189	3.5350	0.0742	0.3008	94.8	100.0
	Branson and Whitehead	2.0643	0.4343	0.4923	1.3670	3.1186	0.0235	0.2430	92.2	100.0
	Walker et al	4.1987	2.1153	1.6132	1.6252	17.0861	2.1579	7.2589	68.2	75.4

**Table 6:** Scenarios 13 & 14: 30% 'good' prognosis, ×3 'good' prognosis survival, 50% and 75% switching probabilities, 100% treatment effect for switchers, uniform distribution for switching times

True HR and *e^ψ^*	Method	Mean estimate	Mean SE	SE of mean	95% Confidence interval		Bias	MSE	Coverage (%)	Successful estimation (%)
					Lower	Upper				
	**Hazard ratio methods**									
	ITT	0.9406	0.0940	0.0965	0.7733	1.1442	0.0406	0.0110	92.2	100.0
	PP - Exclude switchers	1.0018	0.1448	0.1472	0.7547	1.3300	0.1018	0.0320	89.6	100.0
	PP - Censor at switch	2.2865	0.3323	0.3282	1.7198	3.0403	1.3865	2.0301	0.0	100.0
	Time-dependent covariate	3.2815	0.4544	0.4520	2.5017	4.3050	2.3815	5.8757	0.0	100.0
	Law and Kaldor	0.9508	0.1230	0.1263	0.7379	1.2251	0.0508	0.0185	93.7	100.0
	Loeys and Goethebeur	0.8548	-	0.2874	0.4414	1.7657	-0.0452	0.0847	93.6	99.9
0.9 & 1.23	**AFT methods**									
	ITT	1.1696	0.2386	0.2446	0.7843	1.7447	-0.0650	0.0640	92.6	100.0
	PP - Exclude switchers	1.0631	0.1448	0.3143	0.5983	1.8922	-0.1715	0.1282	90.0	100.0
	PP - Censor at switch	0.2100	0.0626	0.0640	0.1172	0.3769	-1.0246	1.0539	0.0	100.0
	Robins and Tsiatis - Logrank	1.2876	-	0.4161	0.6611	2.3440	0.0530	0.1759	94.4	100.0
	Robins and Tsiatis - Cox	1.2835	-	0.4156	0.6581	2.3451	0.0489	0.1751	94.4	95.9
	Robins and Tsiatis - Exponential	1.3120	-	0.3923	0.9033	2.0554	0.0774	0.1599	84.0	99.7
	Robins and Tsiatis - Weibull	1.2898	-	0.4125	0.6781	2.3512	0.0553	0.1732	94.5	99.8
	Branson and Whitehead	1.2789	0.2613	0.3755	0.8571	1.9090	0.0443	0.1429	82.6	100.0
	Walker et al	2.6653	0.9576	0.9545	1.3297	5.5524	1.4307	2.9579	46.7	99.9

	**Hazard ratio methods**									
	ITT	0.8109	0.0842	0.0843	0.6616	0.9939	0.1109	0.0194	71.4	100.0
	PP - Exclude switchers	0.7834	0.1147	0.1158	0.5880	1.0437	0.0834	0.0204	89.7	100.0
	PP - Censor at switch	1.7695	0.2612	0.2559	1.3250	2.3634	1.0695	1.2092	0.0	100.0
	Time-dependent covariate	2.5841	0.3613	0.3598	1.9647	3.3991	1.8841	3.6791	0.0	100.0
	Law and Kaldor	0.8147	0.1113	0.1125	0.6233	1.0649	0.1147	0.0258	81.4	100.0
	Loeys and Goethebeur	0.5287	-	0.1897	0.2352	1.0444	-0.1713	0.0653	86.9	96.9
0.7 & 2.04	**AFT methods**									
	ITT	1.5826	0.3351	0.3379	1.0452	2.3973	-0.4582	0.3242	72.8	100.0
	PP - Exclude switchers	1.7560	0.1147	0.5427	0.9817	3.1462	-0.2849	0.3757	90.5	100.0
	PP - Censor at switch	0.3494	0.1032	0.1020	0.1961	0.6240	-1.6914	2.8712	0.0	100.0
	Robins and Tsiatis - Logrank	2.1469	-	0.7376	1.0853	4.0598	0.1061	0.5552	95.7	100.0
	Robins and Tsiatis - Cox	2.1363	-	0.7256	1.0640	4.0757	0.0955	0.5356	95.9	92.4
	Robins and Tsiatis - Exponential	2.1549	-	0.7419	1.3002	3.6453	0.1140	0.5635	88.4	100.0
	Robins and Tsiatis - Weibull	2.1472	-	0.7359	1.0961	4.0901	0.1064	0.5528	95.6	100.0
	Branson and Whitehead	2.0328	0.4340	0.6210	1.3380	3.0899	-0.0080	0.3857	82.6	100.0
	Walker et al	4.2491	1.5202	1.2616	2.1208	8.6357	2.2083	6.4681	45.3	88.3

**Figure 1:**
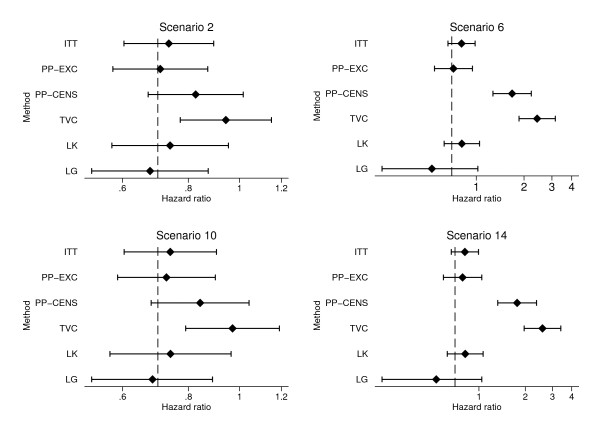
**Mean estimates and confidence limits for adjusted hazard ratio methods from Scenarios 2, 6, 10 and 14**. Note: Mean upper confidence limits truncated at *β *= 4. Vertical lines show true treatment effect (*β *= 0.7).

**Figure 2:**
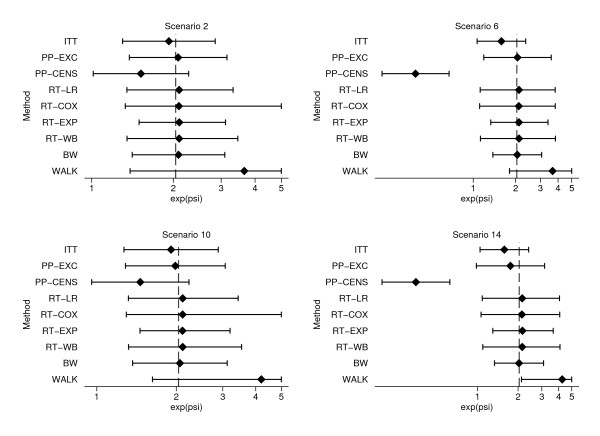
**Mean estimates and confidence limits for AFT methods from Scenarios 2, 6, 10 and 14**. Note: Mean upper confidence limits truncated at *e^ψ ^*= 5 Vertical lines show true treatment effect (*e^ψ ^*= 2.04).

Figure 1 shows mean estimates and mean upper and lower confidence intervals for four simple methods (ITT, PP-EXC, PP-CENS, and TVC) and two adjusted hazard ratio methods (LK, LG). Figure 2 shows mean estimates and mean upper and lower confidence intervals for three simple methods (ITT, PP-EXC, PP-CENS) and for six accelerated failure time model methods (RT-LR, RT-COX, RT-EXP, RT-WB, BW, and WALK).

As expected, the ITT approach underestimated the true treatment effect in each of these four scenarios. This under-estimation was relatively small in the scenarios with a small proportion of switchers (2 and 10), around 0.03 - 0.04 on the hazard ratio scale in both cases. This increased to around 0.11 in scenarios 6 and 14 with a large proportion of control patients switching.

Excluding switchers from the analysis produced relatively small bias in scenarios 2, 6 and 10. However, in scenario 14, where the difference between 'good' and 'poor' prognosis groups and the proportion of switchers were both large, significant bias was seen (0.08 on the hazard ratio scale). The results from this approach are perhaps better than expected with many estimates very close to the true treatment effect, particularly in scenarios where only a small proportion of patients switch treatments. This is possibly explained by the fact that patients who switch treatments have a number of mechanisms acting on them which might cancel each other out. This will be investigated further by comparing biases in scenarios with a smaller and larger true treatment effect in the next section.

Perhaps the most striking results from these scenarios relate to the methods which give particularly large biases, suggesting they are very sensitive to the differences in prognosis between switchers and non-switchers. Of the hazard ratio methods, censoring patients at the time of switching and considering treatment as a time-dependent covariate both produced large biases, particularly when a large proportion of patients switched treatments (Scenarios 6 and 14) with mean hazard ratio estimates of 1.68 and 1.77 for censoring at switch and 2.42 and 2.58 for treatment as a time-varying covariate. These large biases are reflective of what was seen throughout the simulation study for these methods and suggest they may be inappropriate for use due their large sensitivity to even a relatively weak relationship between switching and prognosis.

The parametric method of Walker et al over-estimated the true treatment effect in all four scenarios presented here. This over-estimation was particularly significant in scenarios with a large difference in survival between 'good' and 'poor' prognosis groups (10 and 14), with mean treatment effects of 4.20 and 4.25 over double the true treatment effect of 2.04.

The Law & Kaldor and Loeys & Goetghebeur methods both gave biased estimates in these four scenarios. These biases were particularly large in scenarios with a high proportion of switchers (6 and 14). The Law & Kaldor method seems to underestimate the true treatment effect in all scenarios which is likely to be due to the way in which the method conditions on future events as described by White [13]. Therefore the assumptions made for this method are not met and biases given are likely to be less predictable for a real dataset. The Loeys & Goetghebeur method consistently overestimates the true treatment effect which is perhaps surprising given the method makes the assumption of all-or-nothing compliance, and therefore assumes that a switching patient receives more of the experimental treatment than they actually do. This means that any positive treatment effect seen will actually be due to a smaller amount of treatment than accounted for by the method, so an underestimation of the true treatment effect might be expected. The Robins and Tsiatis method when used with all tests gave very similar mean estimates of *e^ψ ^*, not differing by any more than 0.02, on the hazard ratio scale, in these four scenarios. In all cases the mean estimate of *e^ψ ^*was greater than the true treatment effect of 2.04, suggesting the method is consistently over-adjusting for treatment switching. The mean upper confidence limits given by the Cox model test based method were erratic, suggesting they were being unduly influenced by a few large values. There were also some estimation problems with this method, particularly in scenario 14 with 7.6% of simulations unsuccessful when estimating either *e^ψ ^*or its upper or lower confidence limits.

Very small biases were observed from the Branson and Whitehead method, less than any other AFT method in scenarios 6, 10 and 14. The method also appears to be very robust to more extreme simulated datasets, with 100% successful estimation. Coverage for this method was lower than expected, as low as 82.6% in scenario 14. However, as discussed previously, standard errors calculated from the final regression in the algorithm tend to be too small, giving unduly narrow confidence intervals and therefore lower coverage.

**Figure 3:**
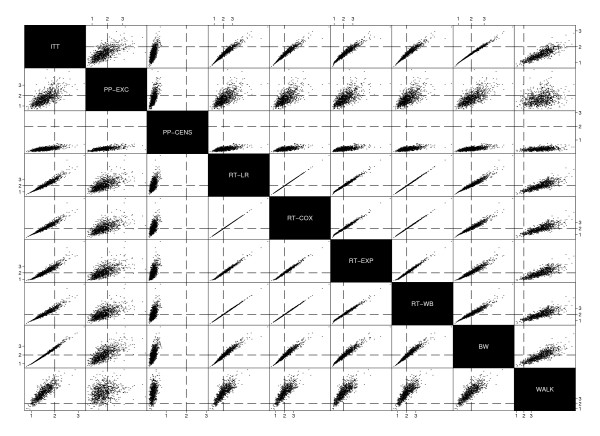
**Scatter plot matrix of hazard ratio method point estimates from Scenario 14**.

**Figure 4:**
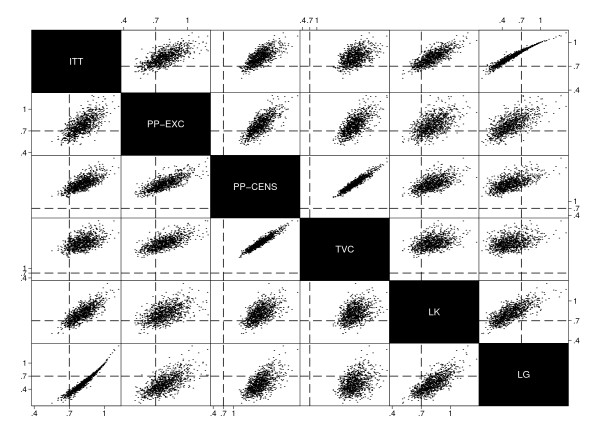
**Scatter plot matrix of AFT method point estimates from Scenario 14**.

The relationships between point estimates from each method in scenario 14 were further investigated through pairwise scatter plots which can be seen in Figures 3 and 4. Vertical and horizontal reference lines show the true treatment effect of *β *= 0.7 for adjusted hazard ratio methods in Figure 3 or *e^ψ ^*= 2.04 for AFT methods in Figure 4.

The relationship between ITT and PP estimates appears to be fairly weak, reflecting the unpredictability of estimates due to biases in this particular scenario. The plots also further illustrate dilution of the true treatment effect when analysing patients as-randomised.

The scatter plot for AFT methods shows the strong relationship between estimates from the Robins & Tsiatis method when using logrank, Cox, exponential or Weibull tests. Relationships between these estimates and those from the Branson & Whitehead method are also strong, although less so than between the Robins & Tsiatis methods themselves. This is to be expected as the model used by Branson & Whitehead takes the same form as that presented by Robins & Tsiatis, differing only by the way in which the estimate of *ψ *is found.

Scatter plots for scenarios 2, 6 and 10 showed similar relationships between parameter estimates.

### Size of true treatment effect

All scenarios focussed on up to this point have had a large true treatment effect (a hazard ratio *β *= 0.7 or *e^ψ ^*= 2.04). As seen previously, biases seen from excluding all switching patients from the analysis were perhaps not as large as expected. The way in which simulated datasets were generated meant that patients who switch treatments should in general have worse prognosis than those who do not, so excluding these patients from the analysis should make the control group have better survival in general and therefore reduce the observed difference between control and experimental groups. However, these switching patients also go on to receive a beneficial treatment, perhaps meaning their survival is approximately similar to the control patients who do not switch treatments. If this was the case, excluding these patients would have a relatively small effect on the estimate of the true treatment effect.

To investigate the competing factors acting upon patients who switch treatments in these simulations, we consider scenarios 9 and 13, which are identical to scenarios 10 and 14 respectively except with a smaller true treatment effect of *β *= 0.9 or *e^ψ ^*= 1.23. Scenarios 9 and 10 have probabilities of 10% and 25% of switching treatments in 'good' and 'poor' prognosis groups whereas 13 and 14 have switching probabilities of 50% and 75%. Full details of these scenarios can be found in Table 2. Full results can be found in Tables 5 and 6.

In general, biases observed were greater in scenarios with a larger true treatment effect than a small effect. A notable exception to this can be seen when comparing scenarios 13 and 14 (Table 6). The bias when excluding switchers was greater in scenario 13 with a small treatment effect. This may be because patients in this scenario who switch treatment have worse prognosis but this is "corrected" to a lesser extent by the treatment they switch onto, making the control arm switchers and non-switchers less similar than in scenario 14 with a larger true treatment effect.

The Branson & Whitehead method also seems to have larger bias in scenarios with a smaller treatment effect. However, these biases are still small, with the mean estimate of *e^ψ ^*closer to the true value than when excluding switchers in both scenarios 13 and 14. There also appears to be a greater difference between estimates given by the various Robins & Tsiatis methods when the true treatment effect is smaller as in scenario 13, although estimates are still strongly related.

### Successful estimation

Most of the methods investigated successfully gave an estimate of the treatment effect in all scenarios. However some of the methods experienced problems in certain situations.

The Walker et al parametric method was particularly unsuccessful in scenarios with a large difference in survival between 'good' and 'poor' prognosis groups and a large true treatment effect, most notably in scenario 12 where the method was successful for only 43.9% of simulated datasets. These problems may have been due to the way the method was implemented in Stata, where attempts to find a maximum likelihood estimate failed to converge. This is further evidence that the method may not be suitable for use, especially given the true treatment effect would not be known in real life.

Some estimation problems were also seen with the Robins & Tsiatis methods when used with a Cox, Weibull or exponential test. Given the similarities between estimates with each test, the logrank test would seem to be the most appropriate choice for this method as it was 100% successful for all scenarios.

### Extension of the Branson & Whitehead method

As seen previously, the method of Branson & Whitehead performed well, giving particularly small biases in scenarios with a large difference in survival between 'good' and 'poor' prognosis groups and a large proportion of switchers, scenarios which other methods gave very biased estimates for (see Tables 4, 5 and 6).

One of the limitations of this method and its practical use is that estimates are given in the AFT model form which is less commonly seen in medical literature than hazard ratios from a proportional hazards model [34]. However, as seen previously if the shape parameter of the Weibull model *γ *is known, hazard ratios can be converted to the AFT parameter *ψ*.

Rearranging (7) gives the following expression for the hazard ratio *β *in terms of *ψ *and *γ *:

(10) β=exp(−γψ)

By taking the value of *γ *estimated in the final iteration of the IPE algorithm, a hazard ratio *β *can be estimated from the method using (10). The standard error of *β *can be calculated using the Delta method as described by Collett [19]. However, these standard errors are likely to be too small as the standard errors of *ψ *and *γ *from which they are calculated are also too small, as described previously. Note that this conversion to a hazard ratio would not be possible for the other AFT methods presented here as they do not directly estimate a shape parameter, *γ*, from the data.

To investigate this extension to the Branson and Whitehead method further, simulations for the scenarios focused on previously (2, 6, 10 and 14) were repeated, with *γ *estimated from the last iteration of the Branson & Whitehead method and used to calculate a hazard ratio and its corresponding standard error as described above. This was compared to hazard ratios from both intention-to-treat and per-protocol approaches for the same simulated data. Table 7 shows mean estimates, bias and the mean standard error for each of the four scenarios.

**Table 7:** Comparison of mean hazard ratios from the Branson & Whitehead method and ITT and PP approaches

Scenario	Method	Mean HR	Bias	Mean SE
**2**	ITT	0.7346	0.0346	0.0734
*(*×1.2 *'Good' prognosis survival, 10% and 25% switching probabilities)*	PP - Exclude switchers	0.7071	0.0071	0.0746
	Branson and Whitehead	0.7077	0.0077	0.0708

**6**	ITT	0.8030	0.1030	0.0808
*(*×1.2 *'Good' prognosis survival, 50% and 75% switching probabilities)*	PP - Exclude switchers	0.7153	0.0153	0.1004
	Branson and Whitehead	0.7172	0.0172	0.0724

**10**	ITT	0.7411	0.0411	0.0763
*(*×3 *'Good' prognosis survival, 10% and 25% switching probabilities)*	PP - Exclude switchers	0.7280	0.0280	0.0793
	Branson and Whitehead	0.7165	0.0165	0.0738

**14**	ITT	0.8121	0.1121	0.0843
*(*×3 *'Good' prognosis survival, 50% and 75% switching probabilities)*	PP - Exclude switchers	0.7810	0.0810	0.1142
	Branson and Whitehead	0.7325	0.0325	0.0762

As seen previously, estimates from the ITT approach are biased towards the null in all four scenarios. This bias is particularly large in scenarios 6 and 14 which have a higher proportion of patients switching from the control arm.

There is very little difference between the mean hazard ratios for the PP and Branson & Whitehead methods in scenarios 2 and 6, with the PP approach giving relatively unbiased estimates due to the small difference in survival between 'good' and 'poor' prognosis patients. However, when this difference is increased in scenarios 10 and 14, the bias from the PP method increases, most notably in scenario 14 where the difference between prognosis groups is coupled with a large proportion of patients switching. The Branson & Whitehead method gives estimates close to the true treatment effect for all four scenarios. The method copes particularly well with the large potential biases in scenario 14, giving a mean hazard ratio of 0.73 compared to 0.78 and 0.81 from the PP and ITT approaches respectively.

The Branson & Whitehead method seems to be robust and to correct for treatment switching most successfully of all methods investigated in situations where a patient's switching pattern is strongly related to their prognosis. The fact that the method can give hazard ratios providing *γ *is estimated from the final iteration of the algorithm is a further advantage if the method were to be more widely used in the analysis of clinical trials.

## Discussion

As expected, adopting an ITT approach underestimated the known treatment effect, most notably in scenarios where a high proportion of patients switched treatments. Results of the ITT analysis are important as they reflect the overall effectiveness of a treatment policy if it were introduced on a wider scale, but in some situations measures of appropriate policy effectiveness are needed in order to answer the relevant policy question.

Commonly adopted approaches of censoring patients at their switching time or considering treatment as a time-dependent covariate were found to be particularly inappropriate, giving biased estimates of the true treatment effect in situations where a patient's switching pattern is strongly related to their underlying prognosis. Excluding switching patients from the analysis altogether gave relatively small biases in situations with a low proportion of switchers, but selection bias increased as switching probabilities increased. Biases from this approach were fairly predictable in this simulation study, but they are likely to be far less so if the approach was applied to real life trials where the underlying prognosis of each patient, and the true treatment effect, are not known.

The Loeys & Goetghebeur method generally gave biased estimates which may be due to the fact that simulations conducted here assumed patients received at least some of their initial treatment, making the "all-or-nothing" assumption inappropriate.

Law & Kaldor's method gave fairly small biases in some scenarios, although the direction of these was difficult to predict. In addition, questions remain about the way in which the method conditions on future events which may bias results towards the null [13,22].

The method of Branson & Whitehead gave the smallest biases of all methods in situations where the potential for selection bias was high. The method performed particularly well when the difference in survival between 'good' and 'poor' prognosis patients was high, which meant patients who switched had worse underlying survival than those who did not. The method was also particularly robust in scenarios with a high proportion of patients who switched, and successfully gave a parameter estimate for all simulated datasets in all of the scenarios presented here. The method did not suffer any convergence problems unlike some of the other methods investigated. It was also demonstrated how the estimates of *e^ψ ^*can be converted to a hazard ratio scale, overcoming one of the main problems with the method being adopted on a wider scale for the analysis of clinical trials with switching patients. In addition, decision models are usually designed in such a way that treatment effects are incorporated using hazard ratios The method of Robins & Tsiatis also gave estimates close to the true treatment effect, but biases were larger than those from the Branson & Whitehead method. The interval bisection method used is more computationally intensive than the IPE algorithm used in the Branson & Whitehead method. Concerns have been raised previously about how the Branson & Whitehead method deals with censoring, with the recensoring used as part of the Robin & Tsiatis method said to be more appropriate [35]. Further investigations into situations with a higher proportion of censored observations are needed.

Problems were seen with the Walker parametric method which gave biased estimates and had estimation problems, most notably in scenarios with a high proportion of switchers. These estimation problems may be due to the way the method was implemented in Stata where convergence to a maximum likelihood estimate was often not achieved. It may be possible for a single dataset to try different initial values and estimating methods, but this was not feasible in our simulation study.

### Limitations

There is a limit to the number of possible scenarios that can be looked at in any simulation study. Clearly there is a need for further simulation work involving many interesting trial variables whose spread of values, individually and in combination could be explored. Most important is the need to assess the performance of methods seen to be successful here in scenarios which violate their model assumptions. It may have been of interest to consider scenarios with even greater potential for selection bias and see how well each method performed. An even greater difference in survival between 'good' and 'poor' prognosis groups could have been introduced which should ensure that patients who switch and those who do not differ greatly in their underlying survival. In addition, the scenarios considered all reflect broadly patients with advanced disease, i.e. events are relatively common; how well the various methods perform in patient populations with less severe disease and therefore fewer events would also be of interest.

Only two true treatment effects were looked at, hazard ratios of 0.9 and 0.7 to represent relatively small and large treatment effects. More values could be investigated, possibly an even larger true effect such as a hazard ratio of 0.5, or a scenario where the treatment which patients were switching onto actually had a negative effect, so a hazard ratio of greater than 1 (or *e^ψ ^<*1). The second of these scenarios would involve a patient's observed survival time being shorter than their underlying event time, a situation in which the recensoring used by the Branson & Whitehead method could be adapted [35].

The method of Branson & Whitehead involves fitting parametric models to the data. In this paper a Weibull distribution was used for this, which allowed the conversion to hazard ratios as described previously. Other parametric distributions commonly used with AFT models could also be used to find an estimate of *ψ *although the same conversion to the hazard ratio scale would not be possible. For example the log-logistic distribution could be used which can deal with non-monotonic hazard functions unlike the Weibull [19]. Branson & Whitehead suggest that a distribution is chosen which best fits the experimental data [23]. A limitation of this work may be that we used a Weibull distribution for our application of the Branson & Whitehead method, and also used a Weibull to generate our simulated data. Potentially, this could make the Branson & Whitehead method appear artificially successful. Further work could be done to investigate how well the method would perform with different choices of distribution, or when applied to data generated from a different distribution.

In simulated datasets patient's switching times were generated from a uniform distribution meaning they were equally likely to switch at any point during their follow-up. This assumption may not be valid in all trial settings and it would be of interest to investigate other switching time distributions, perhaps where the probability of switching is expressed as a function of time since randomisation.

As discussed previously, standard errors given from the last iteration of the IPE algorithm in the Branson & Whitehead method may be too small, with bootstrapping required to give standard errors of the correct size. Given the large number of scenarios considered, and the fact that each of these required 1000 simulations, it was not possible to perform bootstrapping for every one of these. An initial investigation into this was made by repeating simulations for scenario 14 (for which the Branson & Whitehead method previously gave a relatively low coverage of 82.6%) with confidence intervals calculated from 100 bootstrapped samples using the normal approximation method. When using bootstrapping coverage improved to 94.1%.

The simulation study presented only considered the situation where patients switch from the control arm to receive experimental treatment. In reality patients may switch in both directions. For example, some patients may suffer severe side-effects from the experimental treatment and be advised to switch to the control arm. The method of Robins & Tsiatis as implemented through the *strbee *program in Stata does allow switches in both directions to be adjusted for. Branson & Whitehead also state their method can be extended to deal with switching in both directions, although this is yet to be implemented. Further investigation could be done into the way these methods perform in this more complex situation.

We have not covered adjusting for baseline covariates, which can be used to control for imbalances between treatment arms (although this is unlikely in large randomised trials) [36]. Differences in baseline covariates may also account for some of the differences in switching pattern between patients, for example patients of a certain age may be more or less likely to switch treatment groups. Adjusting for these baseline covariates could therefore reduce the biases seen when using some of the simple methods. Branson & Whitehead describe how their method is easily extended by simply including variables in the models fitted as part of the IPE algorithm. Investigations could be performed into this and the extent to which adjusting for baseline covariates can reduce the selection bias observed from the simple methods.

All methods presented give one overall treatment effect and are therefore not necessarily suitable in situations where the treatment effect for patients who switch onto a treatment is not the same as for those who were initially allocated to the experimental treatment arm. This may be particularly important in disease areas such as cancer where treatment switching typically occurs upon disease progression. For example, a recent NICE appraisal of treatments for colorectal cancer [37] found treatment to be around half as effective for patients who switched onto the treatment compared to those who received it from the start of the trial. To properly deal with this situation, new methodology may be needed which gives two different estimates of treatment effect dependent on the time from randomisation or stage of disease at commencement of treatment.

Further methods for dealing with treatment switching which have been published in medical literature were not investigated. A large body of work into causal inference to adjust for post-treatment variables (of which treatment switching would be a special case) has been conducted [38], which may merit further investigation. Hernan et al [39] put forward a method in which patients are censored at the point of their treatment switch but then use inverse probability weighting to adjust for the selection bias this may introduce. Shao et al [40] build on the work of Branson and Whitehead by allowing the causal effect of treatment to differ between patients, although concerns have been raised about their method of estimation [35]. Further investigation may be needed to compare these methods with those presented in this paper.

A recent simulation study by Odondi and McNamee [41] also compared methods for adjusting for non-random complicance, including the Loeys & Goetghebeur and Robins & Tsiatis methods considered here. They concluded that all the methods they considered gave small biases, with the Robins & Tsiatis method performing the best in terms of bias and coverage. However their study differs from ours in the way data were simulated and in some of the outcome measures considered.

Another approach to the analysis of a trial of this sort would be to make use of any external information there is about a treatment. Patients in the control group who switch treatments could have their survival adjusted using this prior information to estimate the survival time they may have experienced if they had not switched. Comparisons between treatment groups could then be made as usual. This would, of course, depend on the availability and quality of external information about the treatment and also the way in which switching had been dealt with in the previous studies, if relevant [42].

## Conclusions

We have illustrated the problem of analysing data from trials in which patients switch treatments and why the ITT approach may not always be sufficient if the appropriate policy effectiveness of a treatment is of interest. The susceptibility of simple methods to selection bias was also seen, particularly if patients who switch treatments were not representative of all patients in the trial.

Given a trial in which a significant proportion of patients switch treatments, a method to adjust for this switching could be used to find an improved estimate of the appropriate policy effectiveness of the treatment. When reporting a trial with treatment crossover, the authors should report the proportion of switchers, a summary of the distribution of switching times and any evidence of a relationship between switching and relevant prognostic variables. Of the methods investigated here, the Branson & Whitehead method gave the smallest bias and was seen to be robust in a variety of scenarios. Further advantages of this method include the conversion of AFT estimates to hazard ratios and its possible extension to trials in which patients switch in both directions between treatment arms, thus easily enabling inclusion of the results into an economic decision model.

## Competing interests

The authors declare that they have no competing interests.

## Authors' contributions

KRA, PCL, AJW and NL conceived the project, and KRA and PCL guided JPM in conducting the project. KRA, AJW and NL sought funding for the study. All authors participated in the design of the simulation study, and JPM carried out the statistical analyses. All authors participated in interpretation of the study results. JPM drafted the paper, which was later revised by all co-authors through substantial contributions to the contents of the paper. All authors read and approved the final version of the manuscript for publication.

## Pre-publication history

The pre-publication history for this paper can be accessed here:

http://www.biomedcentral.com/1471-2288/11/4/prepub

## Supplementary Material

Additional file 1 **A pdf file containing Table A1: Results of Scenarios 3 and 4**. Click here for file

Additional file 2 **A pdf file containing Table A2: Results of Scenarios 7 and 8**. Click here for file

Additional file 3 **A pdf file containing Table A3: Results of Scenarios 11 and 12**. Click here for file

Additional file 4 **A pdf file containing Table A4: Results of Scenarios 15 and 16**. Click here for file
