# Supplementary material for: Assessing methods for dealing with treatment switching in randomised controlled trials: a simulation study
Source: BMC Med Res Methodol. 2011 Jan 11;11:4. doi: 10.1186/1471-2288-11-4 (PMC3024998; doi:10.1186/1471-2288-11-4)
Supplement: Additional file 1 — A pdf file containing Table A1: Results of Scenarios 3 and 4. [file 1471-2288-11-4-S1.PDF]

Additional file 1

Table A1- Results of scenarios 3 and 4

True HR and e^{Ψ}	Method	Mean estimate	Mean SE	SE of mean	95% Confidence interval		Bias	MSE	Coverage (%)	Successful estimation (%)
					Lower	Upper				
	**Hazard ratio methods**									
	ITT	0.9095	0.0888	0.0847	0.7511	1.1013	0.0095	0.0073	96.4	100.0
	PP - Exclude switchers	0.9061	0.0918	0.0868	0.7429	1.1052	0.0061	0.0076	96.8	100.0
	PP - Censor at switch	0.9997	0.1014	0.0959	0.8195	1.2194	0.0997	0.0191	83.8	100.0
	Time-dependent covariate	1.0905	0.1075	0.1037	0.8990	1.3229	0.1905	0.0470	52.5	100.0
	Law and Kaldor	0.9147	0.1146	0.1110	0.7156	1.1692	0.0147	0.0125	96.1	100.0
	Loeys and Goethebeur	0.8948	-	0.0971	0.7159	1.1200	-0.0052	0.0095	96.5	100.0
	**AFT methods**									
0.9 & 1.23	ITT	1.2409	0.2429	0.2328	0.8456	1.8216	0.0063	0.0542	96.9	100.0
	PP - Exclude switchers	1.2522	0.0918	0.2419	0.8411	1.8649	0.0176	0.0588	96.9	100.0
	PP - Censor at switch	1.0277	0.2064	0.1959	0.6935	1.5237	-0.2068	0.0811	83.5	100.0
	Robins and Tsiatis - Logrank	1.2634	-	0.2577	0.8302	1.9283	0.0289	0.0673	96.7	100.0
	Robins and Tsiatis - Cox	1.4663	-	6.3255	0.9554	2.2716	0.2318	40.0651	96.7	95.6
	Robins and Tsiatis - Exponential	1.2683	-	0.2540	0.9692	1.7695	0.0338	0.0657	86.1	99.5
	Robins and Tsiatis - Weibull	1.2632	-	0.2574	0.8346	1.9790	0.0286	0.0671	96.8	99.4
	Branson and Whitehead	1.2618	0.2471	0.2523	0.8597	1.8524	0.0272	0.0644	95.2	100.0
	Walker et al	2.3423	1.4523	1.3770	0.7698	1.38E+24	1.1077	3.1231	79.3	96.6
	**Hazard ratio methods**									
	ITT	0.7233	0.0727	0.0742	0.5939	0.8809	0.0233	0.0060	93.5	100.0
	PP - Exclude switchers	0.7047	0.0734	0.0746	0.5746	0.8642	0.0047	0.0056	94.5	100.0
	PP - Censor at switch	0.7780	0.0811	0.0828	0.6342	0.9543	0.0780	0.0129	83.6	100.0
	Time-dependent covariate	0.8523	0.0862	0.0893	0.6990	1.0391	0.1523	0.0312	51.4	100.0
	Law and Kaldor	0.7250	0.0950	0.0955	0.5608	0.9373	0.0250	0.0098	93.9	100.0
	Loeys and Goethebeur	0.6851	-	0.0820	0.5409	0.8671	-0.0149	0.0069	95.3	100.0
0.7 & 2.04	**AFT methods**									
	ITT	1.9739	0.3992	0.4128	1.3282	2.9345	-0.0669	0.1749	93.7	100.0
	PP - Exclude switchers	2.0811	0.4349	0.4472	1.3819	3.1353	0.0403	0.2016	94.9	100.0
	PP - Censor at switch	1.6964	0.3489	0.3609	1.1338	2.5391	-0.3444	0.2489	82.0	100.0
	Robins and Tsiatis - Logrank	2.0957	-	0.4861	1.3639	3.2876	0.0549	0.2393	95.1	100.0
	Robins and Tsiatis - Cox	2.0918	-	0.4849	1.3481	3.3740	0.0510	0.2378	95.2	93.0
	Robins and Tsiatis - Exponential	2.0975	-	0.4902	1.5012	3.0733	0.0566	0.2435	86.9	100.0
	Robins and Tsiatis - Weibull	2.0955	-	0.4855	1.3635	3.3928	0.0547	0.2387	95.3	100.0
	Branson and Whitehead	2.0842	0.4217	0.4649	1.4022	3.0990	0.0434	0.2180	93.4	100.0
	Walker et al	3.5994	2.2147	1.7570	1.1919	433.6559	1.5586	5.5163	83.7	88.1
