# Supplementary material for: Assessing methods for dealing with treatment switching in randomised controlled trials: a simulation study
Source: BMC Med Res Methodol. 2011 Jan 11;11:4. doi: 10.1186/1471-2288-11-4 (PMC3024998; doi:10.1186/1471-2288-11-4)
Supplement: Additional file 2 — A pdf file containing Table A2: Results of Scenarios 7 and 8. [file 1471-2288-11-4-S2.PDF]

Additional file 2

Table A2 - Results of scenarios 7 and 8

True HR and e^{Ψ}	Method	Mean estimate	Mean SE	SE of mean	95% Confidence interval		Bias	MSE	Coverage (%)	Successful estimation (%)
					Lower	Upper				
	**Hazard ratio methods**									
	ITT	0.9331	0.0912	0.0952	0.7703	1.1302	0.0331	0.0102	92.3	100.0
	PP - Excluding switchers	0.9145	0.1145	0.1162	0.7155	1.1688	0.0145	0.0137	94.9	100.0
	PP - Censor at switch	1.6881	0.2122	0.2132	1.3196	2.1597	0.7881	0.6666	0.1	100.0
	Time-dependent covariate	2.2733	0.2704	0.2713	1.8005	2.8703	1.3733	1.9595	0.0	100.0
	Law and Kaldor	0.9382	0.1179	0.1210	0.7333	1.2003	0.0382	0.0161	93.9	100.0
	Loeys and Goethebeur	0.8652	-	0.2088	0.5520	1.4122	-0.0348	0.0448	92.6	100.0
	**AFT methods**									
0.9 & 1.23	ITT	1.1846	0.2318	0.2438	0.8074	1.7386	-0.0500	0.0619	92.5	100.0
	PP - Excluding switchers	1.2548	0.1145	0.3259	0.7694	2.0481	0.0202	0.1066	95.2	100.0
	PP - Censor at switch	0.3807	0.0946	0.0965	0.2340	0.6200	-0.8539	0.7384	0.0	100.0
	Robins and Tsiatis - Logrank	1.2791	-	0.3729	0.7230	2.1718	0.0445	0.1410	93.6	100.0
	Robins and Tsiatis - Cox	1.2763	-	0.3695	0.7187	2.1811	0.0417	0.1383	93.8	96.4
	Robins and Tsiatis - Exponential	1.2925	-	0.3577	0.9264	1.9155	0.0579	0.1313	82.6	100.0
	Robins and Tsiatis - Weibull	1.2793	-	0.3726	0.7321	2.1830	0.0448	0.1409	93.1	100.0
	Branson and Whitehead	1.2757	0.2498	0.3499	0.8692	1.8728	0.0411	0.1241	83.7	100.0
	Walker et al	2.1688	0.8430	0.8678	1.0268	12.4920	0.9342	1.6258	68.6	100.0
	**Hazard ratio methods**									
	ITT	0.7911	0.0801	0.0799	0.6487	0.9648	0.0911	0.0147	78.8	100.0
	PP - Excluding switchers	0.7115	0.0907	0.0894	0.5542	0.9135	0.0115	0.0081	95.8	100.0
	PP - Censor at switch	1.3084	0.1679	0.1613	1.0174	1.6827	0.6084	0.3962	0.0	100.0
	Time-dependent covariate	1.7865	0.2159	0.2074	1.4097	2.2641	1.0865	1.2235	0.0	100.0
	Law and Kaldor	0.7941	0.1053	0.1043	0.6123	1.0297	0.0941	0.0197	85.8	100.0
	Loeys and Goethebeur	0.5872	-	0.1409	0.3547	0.9471	-0.1128	0.0326	87.6	100.0
0.7 & 2.04	**AFT methods**									
	ITT	1.6499	0.3358	0.3436	1.1073	2.4592	-0.3909	0.2709	78.3	100.0
	PP - Excluding switchers	2.0717	0.5291	0.5357	1.2564	3.4192	0.0309	0.2880	95.5	100.0
	PP - Censor at switch	0.6217	0.1551	0.1499	0.3814	1.0143	-1.4191	2.0364	0.0	100.0
	Robins and Tsiatis - Logrank	2.1084	-	0.6375	1.1770	3.7068	0.0676	0.4110	95.0	100.0
	Robins and Tsiatis - Cox	2.1030	-	0.6356	1.1657	27.3214	0.0622	0.4079	95.3	92.8
	Robins and Tsiatis - Exponential	2.1095	-	0.6432	1.3642	3.3313	0.0687	0.4184	86.8	100.0
	Robins and Tsiatis - Weibull	2.1079	-	0.6375	1.1798	3.7363	0.0671	0.4109	94.9	100.0
	Branson and Whitehead	2.0504	0.4189	0.5733	1.3741	3.0607	0.0095	0.3288	85.3	100.0
	Walker et al	3.6622	1.4002	1.2856	1.7506	12.4920	1.6214	4.2816	68.6	96.4
