# Supplementary material for: Assessing methods for dealing with treatment switching in randomised controlled trials: a simulation study
Source: BMC Med Res Methodol. 2011 Jan 11;11:4. doi: 10.1186/1471-2288-11-4 (PMC3024998; doi:10.1186/1471-2288-11-4)
Supplement: Additional file 3 — A pdf file containing Table A3: Results of Scenarios 11 and 12. [file 1471-2288-11-4-S3.PDF]

Additional file 3

Table A3 - Results of scenarios 11 and 12

True HR and e^{Ψ}	Method	Mean estimate	Mean SE	SE of mean	95% Confidence interval		Bias	MSE	Coverage (%)	Successful estimation (%)
					Lower	Upper				
	**Hazard ratio methods**									
	ITT	0.9137	0.0960	0.1000	0.7437	1.1227	0.0137	0.0102	94.3	100.0
	PP - Excluding switchers	0.9229	0.1008	0.1050	0.7450	1.1433	0.0229	0.0116	94.1	100.0
	PP - Censor at switch	1.0141	0.1108	0.1158	0.8186	1.2563	0.1141	0.0264	80.2	100.0
	Time-dependent covariate	1.1157	0.1183	0.1255	0.9064	1.3735	0.2157	0.0623	50.1	100.0
	Law and Kaldor	0.9237	0.1285	0.1373	0.7032	1.2133	0.0237	0.0194	92.6	100.0
	Loeys and Goethebeur	0.9004	-	0.1148	0.7063	1.1465	0.0004	0.0132	94.8	100.0
	**AFT methods**									
0.9 & 1.23	ITT	1.2449	0.2656	0.2785	0.8196	1.8918	0.0103	0.0777	94.6	100.0
	PP - Excluding switchers	1.2226	0.1008	0.2808	0.7922	1.8876	-0.0120	0.0790	94.2	100.0
	PP - Censor at switch	1.0101	0.2207	0.2318	0.6584	1.5505	-0.2245	0.1041	80.7	100.0
	Robins and Tsiatis - Logrank	1.2748	-	0.3157	0.7982	2.0423	0.0402	0.1013	94.4	100.0
	Robins and Tsiatis - Cox	1.2623	-	0.3137	0.7866	146.2442	0.0277	0.0992	94.7	90.2
	Robins and Tsiatis - Exponential	1.2843	-	0.3054	0.9424	1.9700	0.0497	0.0957	87.2	98.8
	Robins and Tsiatis - Weibull	1.2758	-	0.3147	0.8072	2.1938	0.0412	0.1008	94.9	98.6
	Branson and Whitehead	1.2655	0.2702	0.2996	0.8329	1.9237	0.0310	0.0907	93.4	100.0
	Walker et al	4.1685	2.0271	19.4703	1.8829	1.85E+12	2.9340	387.7004	51.3	88.1
	**Hazard ratio methods**									
	ITT	0.7288	0.0795	0.0819	0.5884	0.9026	0.0288	0.0075	92.8	100.0
	PP - Excluding switchers	0.7209	0.0814	0.0836	0.5778	0.8996	0.0209	0.0074	94.0	100.0
	PP - Censor at switch	0.7917	0.0895	0.0911	0.6344	0.9881	0.0917	0.0167	81.7	100.0
	Time-dependent covariate	0.8770	0.0959	0.0968	0.7078	1.0865	0.1770	0.0407	47.5	100.0
	Law and Kaldor	0.7327	0.1073	0.1066	0.5500	0.9762	0.0327	0.0124	94.9	100.0
	Loeys and Goethebeur	0.6922	-	0.0919	0.5325	0.8965	-0.0078	0.0085	95.1	100.0
0.7 & 2.04	**AFT methods**									
	ITT	1.9753	0.4413	0.4603	1.2753	3.0614	-0.0655	0.2162	93.3	100.0
	PP - Excluding switchers	2.0225	0.4671	0.4869	1.2867	3.1814	-0.0183	0.2374	94.2	100.0
	PP - Censor at switch	1.6586	0.3758	0.3860	1.0641	2.5868	-0.3822	0.2951	81.3	100.0
	Robins and Tsiatis - Logrank	2.1107	-	0.5530	1.3066	3.5262	0.0699	0.3107	94.3	100.0
	Robins and Tsiatis - Cox	2.1057	-	0.5463	1.2480	200.6915	0.0649	0.3026	94.9	88.4
	Robins and Tsiatis - Exponential	2.1121	-	0.5551	1.4217	3.5322	0.0713	0.3132	89.1	100.0
	Robins and Tsiatis - Weibull	2.1105	-	0.5532	1.3076	3.9474	0.0697	0.3109	94.3	100.0
	Branson and Whitehead	2.0777	0.4652	0.5144	1.3400	3.2234	0.0368	0.2659	91.9	100.0
	Walker et al	4.0367	2.7512	1.5027	1.2204	5.99E+18	1.9959	6.2417	85.0	43.9
