# Supplementary material for: Assessing methods for dealing with treatment switching in randomised controlled trials: a simulation study
Source: BMC Med Res Methodol. 2011 Jan 11;11:4. doi: 10.1186/1471-2288-11-4 (PMC3024998; doi:10.1186/1471-2288-11-4)
Supplement: Additional file 4 — A pdf file containing Table A4: Results of Scenarios 15 and 16. [file 1471-2288-11-4-S4.PDF]

**Additional file 4**

**Table A4 - Results of scenarios 15 and 16**

| True HR and $e^{\Psi}$ | Method | Mean estimate | Mean SE | SE of mean | 95% Confidence interval | | Bias | MSE | Coverage (%) | Successful estimation (%) |
|---|---|---|---|---|---|---|---|---|---|---|
| | | | | | Lower | Upper | | | | |
| | **Hazard ratio methods** | | | | | | | | | |
| | ITT | 0.9332 | 0.0983 | 0.0980 | 0.7591 | 1.1472 | 0.0332 | 0.0107 | 94.2 | 100.0 |
| | PP - Excluding switchers | 0.9584 | 0.1300 | 0.1287 | 0.7347 | 1.2503 | 0.0584 | 0.0200 | 93.8 | 100.0 |
| | PP - Censor at switch | 1.7377 | 0.2366 | 0.2300 | 1.3308 | 2.2692 | 0.8377 | 0.7546 | 0.0 | 100.0 |
| | Time-dependent covariate | 2.3775 | 0.3061 | 0.3018 | 1.8473 | 3.0602 | 1.4775 | 2.2741 | 0.0 | 100.0 |
| | Law and Kaldor | 0.9434 | 0.1317 | 0.1349 | 0.7175 | 1.2404 | 0.0434 | 0.0201 | 93.3 | 100.0 |
| | Loeys and Goethebeur | 0.8628 | - | 0.2139 | 0.5160 | 1.4247 | -0.0372 | 0.0471 | 95.0 | 100.0 |
| | **AFT methods** | | | | | | | | | |
| 0.9 & 1.23 | ITT | 1.1903 | 0.2551 | 0.2605 | 0.7823 | 1.8121 | -0.0442 | 0.0698 | 94.1 | 100.0 |
| | PP - Excluding switchers | 1.1522 | 0.1300 | 0.3253 | 0.6736 | 1.9734 | -0.0824 | 0.1126 | 94.0 | 100.0 |
| | PP - Censor at switch | 0.3582 | 0.0974 | 0.0986 | 0.2103 | 0.6109 | -0.8764 | 0.7777 | 0.0 | 100.0 |
| | Robins and Tsiatis - Logrank | 1.2915 | - | 0.4067 | 0.6867 | 2.3459 | 0.0569 | 0.1686 | 95.0 | 100.0 |
| | Robins and Tsiatis - Cox | 1.2895 | - | 0.3997 | 0.6793 | 26.1962 | 0.0550 | 0.1628 | 95.5 | 91.4 |
| | Robins and Tsiatis - Exponential | 1.3123 | - | 0.3846 | 0.9009 | 2.1561 | 0.0777 | 0.1539 | 89.0 | 99.5 |
| | Robins and Tsiatis - Weibull | 1.2934 | - | 0.4038 | 0.7018 | 2.4303 | 0.0588 | 0.1665 | 95.0 | 99.6 |
| | Branson and Whitehead | 1.2833 | 0.2754 | 0.3688 | 0.8428 | 1.9549 | 0.0487 | 0.1384 | 87.4 | 100.0 |
| | Walker et al | 3.2100 | 1.2199 | 1.1532 | 1.5455 | 12.4920 | 1.9754 | 5.2321 | 33.7 | 98.5 |
| | **Hazard ratio methods** | | | | | | | | | |
| | ITT | 0.7935 | 0.0873 | 0.0918 | 0.6395 | 0.9846 | 0.0935 | 0.0172 | 79.4 | 100.0 |
| | PP - Excluding switchers | 0.7567 | 0.1049 | 0.1069 | 0.5766 | 0.9930 | 0.0567 | 0.0146 | 91.7 | 100.0 |
| | PP - Censor at switch | 1.3605 | 0.1898 | 0.1917 | 1.0351 | 1.7884 | 0.6605 | 0.4730 | 0.0 | 100.0 |
| | Time-dependent covariate | 1.8960 | 0.2483 | 0.2504 | 1.4667 | 2.4510 | 1.1960 | 1.4930 | 0.0 | 100.0 |
| | Law and Kaldor | 0.7927 | 0.1175 | 0.1185 | 0.5929 | 1.0601 | 0.0927 | 0.0226 | 87.5 | 100.0 |
| | Loeys and Goethebeur | 0.5883 | - | 0.1712 | 0.3185 | 0.9983 | -0.1117 | 0.0418 | 88.7 | 99.6 |
| 0.7 & 2.04 | **AFT methods** | | | | | | | | | |
| | ITT | 1.6640 | 0.3733 | 0.3939 | 1.0723 | 2.5835 | -0.3769 | 0.2972 | 81.2 | 100.0 |
| | PP - Excluding switchers | 1.8641 | 0.5229 | 0.5431 | 1.0765 | 3.2326 | -0.1767 | 0.3262 | 91.5 | 100.0 |
| | PP - Censor at switch | 0.5829 | 0.1584 | 0.1626 | 0.3425 | 0.9935 | -1.4579 | 2.1520 | 0.0 | 100.0 |
| | Robins and Tsiatis - Logrank | 2.1427 | - | 0.7352 | 1.1205 | 4.0697 | 0.1019 | 0.5510 | 93.9 | 100.0 |
| | Robins and Tsiatis - Cox | 2.4803 | - | 9.8483 | 1.2768 | 54.9580 | 0.4394 | 97.1822 | 94.3 | 86.6 |
| | Robins and Tsiatis - Exponential | 2.1439 | - | 0.7441 | 1.2881 | 3.8447 | 0.1031 | 0.5644 | 86.9 | 99.8 |
| | Robins and Tsiatis - Weibull | 2.1432 | - | 0.7368 | 1.1272 | 4.2830 | 0.1023 | 0.5534 | 94.1 | 99.8 |
| | Branson and Whitehead | 2.0437 | 0.4625 | 0.6392 | 1.3119 | 3.1855 | 0.0029 | 0.4086 | 85.5 | 100.0 |
| | Walker et al | 4.2789 | 1.7206 | 1.1840 | 1.9790 | 12.4920 | 2.2380 | 6.4107 | 49.9 | 56.1 |
